# Association of Urinary Sodium Excretion and Diabetic Kidney Disease in Patients With Type 2 Diabetes Mellitus: A Cross-Sectional Study

**DOI:** 10.3389/fendo.2021.772073

**Published:** 2021-10-28

**Authors:** Yan Huang, Wenhui Liu, Jianfang Liu, Dan Guo, Peizhen Zhang, Deying Liu, Jiayang Lin, Linjie Yang, Huijie Zhang, Yaoming Xue

**Affiliations:** ^1^ Department of Endocrinology and Metabolism, Nanfang Hospital, Southern Medical University, Guangzhou, China; ^2^ Guangdong Provincial Key Laboratory of Shock and Microcirculation, Guangzhou, China; ^3^ Department of Food Safety and Health Research Center, School of Public Health, Southern Medical University, Guangzhou, China

**Keywords:** diabetic kidney disease, type 2 diabetes, urinary sodium excretion, insulin resistance, vascular sclerosis

## Abstract

**Background:**

Diabetic kidney disease (DKD) is the leading cause of end-stage kidney disease worldwide. Epidemiological evidence of the association between urinary sodium excretion and the presence of DKD in patients with type 2 diabetes mellitus (T2DM) has not yet been well established.

**Methods:**

We performed a cross-sectional study of 1545 patients with T2DM over aged 20 years old from January 2018 to December 2020. Urinary sodium excretion was measured by 24-hour urine samples in inpatients and morning fasting urine samples in outpatients. The associations between urinary sodium excretion and the risks of DKD were examined using stepwise regression analysis, logistic regression analysis and multivariable-adjusted restricted cubic splines (RCS).

**Results:**

Regression analysis showed that urinary sodium was independently associated with urinary albumin to creatinine ratio (UACR) level (*P* = 0.006) and the risks of DKD (*P* = 0.042). In multivariable-adjusted RCS analysis, urinary sodium excretion was significantly associated with UACR in all patients (*P* = 0.008), and exhibited a J-shaped relationship. Logistic regression analysis showed that increased urinary sodium excretion was significantly associated with increased risks of DKD [OR (95% CI); 1.56 (1.07-2.27); *P* = 0.020]. However, the relationships between urinary sodium excretion and the risks of DKD and albuminuria showed no significance, after further adjustment for HOMA-IR and ba-PWV (brachial-ankle pulse wave velocity) (Both *P* > 0.05).

**Conclusions:**

Higher urinary sodium excretion level was associated with increased risks of DKD among patients with T2DM, dependent of vascular sclerosis and insulin resistance.

## Introduction

Diabetic kidney disease (DKD), one of the common complications of diabetes mellitus, was strongly associated with all-cause and cardiovascular disease (CVD) mortality in a multiethnic Asian population (1). DKD is the leading cause of end-stage kidney disease worldwide, accounting for approximately 50% of cases in the developed countries (2). The World Health Organization (WHO) multinational study showed that renal disease including DKD accounted for 11% of all deaths of patients with type 2 diabetes mellitus (T2DM) (3). In China, approximately 24.3 million have DKD and 60.5% of patients with diabetes have reduced kidney function or slightly increased albuminuria (4).

It has been well documented that sodium intake is positively associated with the risks of clinical CVD events and chronic kidney disease (CKD) (5, 6). However, the association between sodium intake and the risks of DKD remains less clear. Albuminuria has emerged as a sensitive marker of kidney damage (7), and a predictive risk factor for end-stage renal failure in diabetic individuals (8). Epidemiological evidence conducted in the general population and patients with type 1 diabetes and reported that sodium intake was positively related to urinary albumin excretion (9, 10), particularly in overweight subjects (10). Additionally, few studies reported a reverse J-shaped or no significant association between dietary sodium and urinary albumin in patients with T2DM (11, 12). Indeed, dietary sodium intake measurement methods may contribute to these conflicting findings. However, these evidences did not provide a conclusive information regarding the association between sodium intake and DKD in patients with T2DM. We quantified urinary sodium excretion by 24-hour urine samples, which be considered the most reliable estimate of sodium intake (13), and aimed to explore the associations between urinary sodium excretion and the risks of DKD in patients with T2DM.

## Materials and Methods

### Study Participants

This cross-sectional study was based on the Nanfang Prospective Diabetes Study (NFPDS), a prospective cohort study designed to explore the associations of urinary electrolyte and possible risk factors of microvascular complications in patients with T2DM from Nanfang Hospital, Southern Medical University. We followed the methods of Liu et al. (14). A total of 1545 inpatients and outpatients over aged 20 years old from Guangzhou city, China, was included in this study from January 2018 to December 2020. In the present study, participants completed urine collections and evaluation of diabetic microvascular complications. Inclusion criteria was diagnosis of T2DM according to the criteria of the American Diabetes Association (ADA) (2017) (15). The individuals undergoing dialysis treatment, being pregnant or planning to become pregnant, and patients with NYHA class III or IV congestive heart failure and severe systemic infection were excluded. All participants completed a uniform questionnaire regarding demographics, lifestyle habits (i.e., smoking status, alcohol consumption) and medical history.

The protocol for the study received ethical approval conforming to the Declaration of Helsinki from the Institutional Review Board of Nanfang Hospital, Southern Medical University. All participants provided written informed consent.

### Measurements

Physical examination included height, weight and blood pressure (BP) were screened following a standardized protocol. The height and weight were measured by using the same automatic measuring instrument. Body mass index (BMI) was calculated as weight (kg) divided by square of height (m^2^). BP was measured in triplicate with an electronic sphygmomanometer (OMRON Company). The mean value of the three readings were used for analysis. ba-PWV (brachial-ankle pulse wave velocity, ba-PWV) was measured using an arteriosclerosis detection device (Omron, BP-203RPEIII).

Overnight fasting blood samples of inpatients and outpatients were obtained and tested in the laboratory of Nanfang hospital with stringent quality control. Triglyceride (TG), total Cholesterol (TC), low-density lipoprotein cholesterol (LDL-c), creatinine (CR), uric acid (UA) was determined by enzymatic methods using a fully automated biochemical analyzer. Urinary creatinine (Ucr), urinary albumin to creatinine ratio (UACR) were determined by automatic protein - specific analyzer (Afinion AS100). Glycated hemoglobin (HbA1c) was determined by high performance liquid chromatography. Fasting plasma glucose concentrations were determined by the hexokinase method. Fasting insulin level was determined by electroluminescent immunoassay. According to the test results, index of homeostasis model assessment of insulin resistance (HOMA-IR) was calculated according to the following formula: HOMA-IR = fasting insulin (mIU/L) ×FBG (mmol/L)/22.5. Inpatients were asked to collect 24-hour urine samples for the measurement of urinary sodium level. Outpatients were asked to collect morning fasting urine samples to measure spot urinary sodium. The Kawasaki formula was used to estimate 24-hour urinary sodium excretion of outpatients, which be considered valid for estimating sodium intake in healthy participants and patients with antihypertensive therapy (16, 17). 24-hour and spot urinary sodium concentrations were measured by ion selective electrode method.

On the basis of the mean of three seated, hypertension was defined as mean BP of 140/90 mmHg or greater and/or the self-reported use of antihypertensive medication. Hyperlipidemia was defined as total cholesterol (TC) ≥ 6.22mmol/l, or low-density lipoprotein cholesterol (LDL-c) ≥ 4.14 mmol/l, or triglycerides (TG) ≥ 2.26 mmol/l and/or self-reported use of lipid-lowering drugs.

### Definitions of Albuminuria and DKD

Albuminuria was diagnosed as UACR ≥ 30 mg/g, while excluding infection and other factors. The value of UACR was obtained by calculating urinary albumin (mg) to creatinine (g) ratio. DKD was diagnosed as UACR ≥ 30 mg/g and/or eGFR < 60 ml·min^-1^· (1.73 m²)^-1^, while excluding other causes of chronic kidney disease. The estimated glomerular filtration rate (eGFR) was calculated by using the CKD-EPI formula (18). Definitions as described above were obtained according to the criteria of ADA (2017) (19).

### Statistical Analysis

Baseline characteristics were described as means ± standard deviation (SD), median (interquartile range), frequencies, or percentage. Data that were not normally distributed were logarithmically transformed before analysis. General linear models (GLM) and the chi-square test were used to compare the differences between the four quartiles of urinary sodium excretion. The stepwise regression was used to determine the relationship between variables and DKD. Multivariate logistics regression model was used to estimate relationship between urinary sodium excretion level and the risk of DKD as well as albuminuria. RCS model was used to investigate the relationship between urinary sodium excretion and UACR level. Forest plot was used to examine the relationship between urinary sodium excretion levels and the risk of DKD in different subgroups. According to the recommendations of WHO (20), urinary sodium excretion of less than 2 g/d was selected as the reference group for all spline plots. Statistical analyses were performed by using SAS version 9.4 (SAS Institute Inc). A two-sided *P* < 0.05 was considered statistically significant.

## Results

**Table 1** presents the clinical characteristics of patients categorized by presence of DKD. The mean age of the subjects with DKD was 56.5 ± 10.6 years. Subjects with DKD had higher urinary sodium excretion level than those with non-DKD (3.42 ± 1.48 g/d *vs*. 3.23 ± 1.42 g/d, respectively, *P* = 0.029). Subjects with DKD exhibited greater age, longer duration of diabetes, higher levels of SBP, DBP, PWV, TG, CR, UA, UACR than those with non-DKD (All *P* < 0.05). Likewise, The DKD group had higher prevalence of hyperlipidemia and hypertension than the non-DKD group. Furthermore, patients with DKD had higher percentage of using RAS blocking agents, diuretics, statin, SGLT 2i (All *P* < 0.05). The level of eGFR was lower in patients with DKD compared to those with non-DKD (*P* < 0.001). There were no significant differences in the levels of BMI, HbA1c, glucose, HOMA-IR, TC and LDL-c between two groups (All *P >* 0.05).

**Table 1:** Characteristics of patients categorized by presence of DKD.

Variables	non-DKD	DKD	*P*-value
Sample size	1209	336	–
Urinary sodium (g/d)	3.23 ± 1.42	3.42 ± 1.48	0.029
Age (years)	54.5 ± 11.0	56.5 ± 10.6	0.004
Gender (Male n, %)	772 (63.9)	219 (65.2)	0.654
Smoking (n, %)	545 (45.1)	167 (50.2)	0.103
Alcohol use (n, %)	398 (33.0)	126 (37.8)	0.095
Duration of diabetes (years)	6 (2-11)	11 (5-16)	<0.001
BMI (kg/m^2^)	24.5 ± 3.5	24.7 ± 3.8	0.290
SBP (mmHg)	125.8 ± 17.5	133.4 ± 19.4	<0.001
DBP (mmHg)	76.7 ± 11.0	79.2 ± 10.7	<0.001
Hypertension (n, %)	353 (29.2)	184 (54.8)	<0.001
Hyperlipidemia (n, %)	652 (53.9)	246 (73.2)	<0.001
Antidiabetic medication (n, %)	847 (70.1)	284 (84.5)	<0.001
RAS blocking agents (n, %)	133 (11.0)	107 (31.9)	<0.001
Diuretics (n, %)	31 (2.6)	17 (5.1)	0.020
Statin (n, %)	132 (10.9)	76 (22.6)	<0.001
SGLT 2i (n, %)	19 (1.6)	13 (3.9)	0.009
PWV (cm/s)	1571.6 ± 311.9	1736.4 ± 346.9	<0.001
HbA1c (%)	9.3 ± 2.6	9.4 ± 2.4	0.579
Glucose (mmol/L)	8.7 ± 4.4	8.7 ± 5.1	0.876
HOMA-IR	0.70 ± 1.01	0.73 ± 1.18	0.667
TG (mmol/l)	1.44 (0.98-2.26)	1.65 (1.08-3.05)	0.003
TC (mmol/l)	5.00 ± 1.30	5.07 ± 1.58	0.381
LDL-c (mmol/l)	3.21 ± 0.94	3.23 ± 0.97	0.712
CR (μmol/l)	66.0 (55.0-78.0)	80.5 (60.0-111.0)	<0.001
UA (μmol/l)	354.16 ± 110.10	377.63 ± 125.79	<0.001
UACR (mg/mmol)	1.1 (0.7-2.6)	9.4 (3.3-46.0)	<0.001
eGFR (ml/min/1.73m²)	95.83 ± 22.47	79.52 ± 31.97	<0.001

BMI, body mass index; SBP, systolic blood pressure; DBP, diastolic blood pressure; RAS, renin-angiotensin system; PWV, pulse wave velocity; HbA1c, glycated hemoglobin; HOMA-IR, homeostasis model assessment of insulin resistance; TG, triglyceride; TC, total cholesterol; LDL-c, low-density lipoprotein cholesterol; CR, creatinine; UA, uric acid; UACR, urinary albumin to creatinine ratio; eGFR, estimated glomerular filtration rat.

The baseline characteristics of participants categorized by quartile of urinary sodium excretion are showed in **Table 2**. The median of duration of diabetes was 7 (2-12) years. Patients in the higher quartiles had higher prevalence of smoking, longer duration of diabetes and higher percentage of using antidiabetic medication than those in the lower quartiles (All *P* < 0.05). In addition, there was a significant increasing trend in the levels of BMI, HOMA-IR and eGFR with increasing urinary sodium excretion. Conversely, there was a significant decreasing trend in the levels of HbA1c, TC and LDL-c with increasing urinary sodium excretion. Levels of SBP, DBP, PWV, TG, UR, UA and UACR, glucose showed no differences among the four quartiles of urinary sodium excretion (All *P* > 0.05). Of note, the prevalence of DKD from the lowest quartile to the highest quartile was 19.7%, 20.0%, 21.0%, 26.4%, respectively (*P* = 0.028), adjusted for age and gender.

**Table 2:** Characteristics of patients categorized by quartile of urinary sodium excretion level.

Variables	Total	Estimated 24-hour urinary sodium excretion level				*P*-value
		Quartile 1	Quartile 2	Quartile 3	Quartile 4	
Sample size	1545	386	386	386	387	–
Urinary sodium (g/d)	3.27 ± 1.44	1.69 ± 0.45	2.72 ± 0.25^b^	3.51 ± 0.25^b^	5.16 ± 1.21^b^	<0.001
Age (years)	54.9 ± 10.9	54.8 ± 11.6	55.5 ± 10.7	55.4 ± 10.7	54.0 ± 10.6	0.226
Gender (Male n, %)	991 (64.1)	218 (56.5)	236 (61.1)	257 (66.6)	280 (72.4)	<0.001
Smoking (n, %)	712 (46.2)	161 (41.7)	166 (43.1)	188 (49.0)	197 (51.0)	0.024
Alcohol use (n, %)	524 (34.0)	116 (30.1)	127 (33.0)	144 (37.5)	137 (35.5)	0.149
Duration of diabetes (years)	7 (2-12)	4 (2-10)	7 (2-12) ^b^	8 (2-13)^b^	7 (2-13) ^b^	<0.001
BMI (kg/m^2^)	24.5 ± 3.5	23.9 ± 3.5	24.4 ± 3.5^a^	24.5 ± 3.1^a^	25.4 ± 3.7^b^	<0.001
SBP (mmHg)	127.4 ± 18.3	126.4 ± 18.4	126.6 ± 18.6	127.9 ± 18.6	128.9 ± 17.4	0.174
DBP (mmHg)	77.2 ± 11.0	77.0 ± 11.7	76.9 ± 10.5	76.7 ± 10.7	78.4 ± 10.9	0.127
Hypertension (n, %)	537 (34.8)	152 (39.4)	131 (33.9)	123 (31.9)	131 (33.9)	0.150
Hyperlipidemia (n, %)	898 (58.1)	227 (58.8)	226 (58.6)	228 (59.1)	217 (56.1)	0.822
Antidiabetic medication (n, %)	1131 (73.2)	238 (61.7)	283 (73.3)	306 (79.3)	304 (78.6)	<0.001
RAS blocking agents (n, %)	240 (15.5)	61 (15.8)	56 (14.5)	67 (17.4)	56 (14.5)	0.650
Diuretics (n, %)	48 (3.1)	18 (4.7)	10 (2.6)	9 (2.3)	11 (2.8)	0.230
Statin (n, %)	208 (13.5)	46 (11.9)	52 (13.5)	57 (14.8)	53 (13.7)	0.712
SGLT 2i (n, %)	32 (2.1)	4 (1.0)	10 (2.6)	7 (1.8)	11 (2.8)	0.282
PWV (cm/s)	1607.1 ± 326.8	1602.0 ± 333.3	1602.7 ± 335.6	1602.7 ± 319.9	1621.3 ± 319.0	0.825
HbA1c (%)	9.4 ± 2.5	10.1 ± 2.8	9.5 ± 2.5^b^	8.9 ± 2.4^b^	8.9 ± 2.2^b^	<0.001
Glucose (mmol/L)	8.7 ± 4.6	8.9 ± 5.4	8.8 ± 4.5	8.3 ± 3.9	8.6 ± 4.3	0.278
HOMA-IR	0.71 ± 1.04	0.46 ± 1.07	0.65 ± 1.07^a^	0.74 ± 1.04^b^	0.99 ± 0.93^b^	<0.001
TG (mmol/l)	1.48 (1.01-2.38)	1.38 (0.95-2.23)	1.52 (0.98-2.42)	1.47 (1.02-2.40)	1.58 (1.04-2.66)	0.427
TC (mmol/l)	5.01 ± 1.37	5.08 ± 1.57	5.14 ± 1.32	4.86 ± 1.26^a^	4.98 ± 1.29	0.023
LDL-c (mmol/l)	3.21 ± 0.95	3.25 ± 1.02	3.33 ± 0.97	3.13 ± 0.95	3.15 ± 0.82	0.013
CR (μmol/l)	68.0 (55.0-83.0)	65.0 (54.0-84.0)	68.0 (55.0-85.0)	70.0 (59.0-85.0)	68.0 (55.0-80.0)	0.708
UA (μmol/l)	359.26 ± 114.06	354.04 ± 115.65	356.31 ± 110.54	364.38 ± 121.77	362.31 ± 107.95	0.548
UACR (mg/mmol)	1.5 (0.7-5.3)	1.6 (0.8-5.0)	1.4 (0.7-6.3)	1.4 (0.7-4.3)	1.5 (0.8-6.5)	0.264
eGFR (ml/min/1.73m²)	92.28 ± 25.73	91.88 ± 26.61	91.60 ± 25.86	90.06 ± 26.36	95.58 ± 23.78^a^	0.022
Diabetic kidney disease (n, %)^c^	336 (21.8)	76 (19.7)	77 (20.0)	81 (21.0)	102 (26.4)^a^	0.028

BMI, body mass index; SBP, systolic blood pressure; DBP, diastolic blood pressure; RAS, renin-angiotensin system; PWV, pulse wave velocity; HbA1c, glycated hemoglobin; HOMA-IR, homeostasis model assessment of insulin resistance; TG, triglyceride; TC, total cholesterol; LDL-c, low-density lipoprotein cholesterol; CR, creatinine; UA, uric acid; UACR, urinary albumin to creatinine ratio; eGFR, estimated glomerular filtration rate.

a P < 0.05 compared with Quartile 1 of urinary sodium.

b P < 0.01 compared with Quartile 1 of urinary sodium.

c Adjusted for age and gender.

As shown in **Table 3**, the levels of PWV, HbA1c, CR, UA and urinary sodium were independently correlated with UACR level and the presence of DKD in stepwise regression analysis (All *P* < 0.05). In addition, age, gender, and the levels of BMI, DBP, SBP, TC were significantly correlated with UACR level.

**Table 3:** Stepwise regression analysis with UACR level and DKD.

Variables	UACR			DKD		
	Regression coefficient β	Standard error	*P*-value	Regression coefficient β	Standard error	*P*-value
Age (years)	-0.013	0.004	0.024	–	–	–
Gender	-0.440	0.091	<0.001	–	–	–
Smoking	–	–	–	–	–	–
Alcohol use	–	–	–	–	–	–
BMI (kg/m^2^)	-0.019	0.012	0.098	–	–	–
SBP (mmHg)	0.023	0.004	<0.001	–	–	–
DBP (mmHg)	-0.009	0.005	0.043	–	–	–
PWV (cm/s)	0.001	0.0002	<0.001	0.001	0.0002	<0.001
HbA1c (%)	0.110	0.016	<0.001	0.086	0.029	0.015
HOMA-IR	–	–	–	–	–	–
TC (mmol/l)	0.125	0.029	<0.001	–	–	–
LDL-c (mmol/l)	–	–	–	–	–	–
CR (μmol/l)	1.032	0.093	<0.001	0.979	0.145	<0.001
UA (μmol/l)	0.001	0.0004	<0.001	0.001	0.0006	0.027
Urinary sodium (g/d)	0.114	0.040	0.006	0.139	0.069	0.042

UACR, urinary albumin to creatinine ratio; DKD, diabetic kidney disease; BMI, body mass index; SBP, systolic blood pressure; DBP, diastolic blood pressure; PWV, pulse wave velocity; HbA1c, glycated hemoglobin; HOMA-IR, homeostasis model assessment of insulin resistance; TC, total cholesterol; LDL-c, low-density lipoprotein cholesterol; CR, creatinine; UA, uric acid.

The forward stepwise regression analysis was used to obtain the determinants of UACR and DKD.

**Figure 1:**
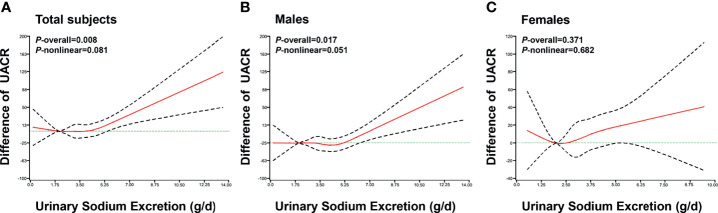
The association of urinary sodium excretion with UACR level. The restricted cubic spline (RCS) regression was used to analyze the relationships of urinary sodium excretion (g/d) with urinary albumin to creatinine ratio (UACR) after adjusting for age, gender, BMI, smoking, alcohol consumption, DBP, RAS blocking agents, diuretics, hyperlipidemia, statin, HbA1c and antidiabetic drugs in total subjects **(A)**, males **(B)**, females **(C)**. Urinary sodium excretion was coded using an RCS function with five knots located at the 5th, 25th, 50th, 75th, 95th percentiles of the distribution of urinary sodium excretion. Y-axis represents the difference in UACR between individuals with any value of urinary sodium excretion with individuals with 2g/d of urinary sodium excretion. X-axis represents the continuous change of urinary sodium excretion. Black dashed lines are 95 percent confidence intervals.

The RCS analysis showed that urinary sodium excretion was significantly associated with UACR level in all patients (*P* = 0.008) and males (*P* = 0.017), after adjusted for age, gender, BMI, smoking, alcohol consumption, DBP, HbA1c, use of RAS blocking agents, diuretics, hyperlipidemia, statin, and antidiabetic drugs (**Figure 1**). However, there was insignificant nonlinear association between urinary sodium excretion and UACR level (*P* = 0.081). A J-shaped relationship was observed between urinary sodium excretion and UACR level.

The multivariable-adjusted odds ratios (ORs) for the association between urinary sodium excretion and the risks of DKD and albuminuria are shown in **Table 4**. After adjustment for age, gender, BMI, smoking, alcohol consumption, DBP, HbA1c, and use of RAS blocking agents, diuretics, hyperlipidemia, statin and antidiabetic medication, individuals in highest quartile of urinary sodium excretion were 1.56 times more likely to have DKD than those in the lowest quartile [OR (95% CI); 1.56 (1.07-2.27); *P* = 0.020]; urinary sodium excretion was significantly associated with the increased risks of DKD (*P* < 0.05). However, the relationship between urinary sodium excretion and the risks of DKD showed no significance, after further adjustment for HOMA-IR and PWV (*P* > 0.05). In the subgroup analyses of the associations between the risk of DKD and urinary sodium excretion levels according to the following variables: age (< 60 years/≥ 60 years), gender (male/female), hypertension (yes/no), BMI (< 24 kg/m^2/^≥ 24 kg/m^2^), duration of diabetes (≤ 5 years/> 5 years), HbA1c (< 9%/≥ 9%), SGLT_2i(yes/no), Diuretics(yes/no), the result indicated that the relationships of sodium excretion levels and the risk of DKD had no interaction between different subgroups (*P*-interaction > 0.05) (**Supplementary Figure 1**).

**Table 4:** Odds ratios (ORs) of DKD and albuminuria according to urinary sodium excretion levels.

Variables	DKD			Albuminuria		
	OR	95% CI	*P*-value	OR	95% CI	*P*-value
**Crude model 1**						
urinary sodium (g/d)	1.09	0.96-1.23	0.189	1.19	1.02-1.41	0.033
urinary sodium (g/d)						
(Quartile 2 *vs*. Quartile 1)	0.84	0.58-1.22	0.362	0.96	0.57-1.61	0.871
(Quartile 3 *vs*. Quartile 1)	0.96	0.67-1.38	0.833	0.87	0.51-1.48	0.595
(Quartile 4 *vs*. Quartile 1)	1.29	0.91-1.82	0.154	1.17	0.71-1.92	0.550
**Crude model 2**						
urinary sodium (g/d)	1.12	0.99-1.27	0.083	1.23	1.04-1.46	0.019
urinary sodium (g/d)						
(Quartile 2 *vs*. Quartile 1)	1.02	0.70-1.49	0.905	1.23	0.70-2.17	0.467
(Quartile 3 *vs*. Quartile 1)	1.05	0.72-1.52	0.805	1.07	0.60-1.92	0.809
(Quartile 4 *vs*. Quartile 1)	1.44	1.00-2.06	0.049	1.53	0.88-2.65	0.129
**Model 1**						
urinary sodium (g/d)	1.13	1.00-1.28	0.045	1.23	1.04-1.45	0.014
urinary sodium (g/d)						
(Quartile 2 *vs*. Quartile 1)	0.98	0.69-1.40	0.906	1.09	0.65-1.81	0.747
(Quartile 3 *vs*. Quartile 1)	1.01	0.71-1.45	0.944	0.93	0.55-1.57	0.781
(Quartile 4 *vs*. Quartile 1)	1.42	1.01-2.01	0.046	1.37	0.83-2.26	0.219
**Model 2**						
urinary sodium (g/d)	1.19	1.04-1.35	0.009	1.24	1.05-1.47	0.014
urinary sodium (g/d)						
(Quartile 2 *vs*. Quartile 1)	1.04	0.72-1.51	0.839	1.10	0.65-1.86	0.724
(Quartile 3 *vs*. Quartile 1)	1.03	0.71-1.50	0.872	0.96	0.56-1.64	0.878
(Quartile 4 *vs*. Quartile 1)	1.62	1.13-2.33	0.009	1.37	0.82-2.31	0.234
**Model 3**						
urinary sodium (g/d)	1.17	1.03-1.34	0.016	1.22	1.03-1.46	0.024
urinary sodium (g/d)						
(Quartile 2 *vs*. Quartile 1)	0.99	0.68-1.45	0.976	1.03	0.61-1.76	0.903
(Quartile 3 *vs*. Quartile 1)	1.02	0.69-1.49	0.926	0.93	0.54-1.61	0.800
(Quartile 4 *vs*. Quartile 1)	1.56	1.07-2.27	0.020	1.28	0.75-2.17	0.373
**Model 4**						
urinary sodium (g/d)	1.13	0.99-1.30	0.076	1.19	0.99-1.43	0.068
urinary sodium (g/d)						
(Quartile 2 *vs*. Quartile 1)	0.98	0.66-1.45	0.914	1.11	0.62-2.00	0.733
(Quartile 3 *vs*. Quartile 1)	0.98	0.66-1.47	0.930	0.98	0.54-1.80	0.959
(Quartile 4 *vs*. Quartile 1)	1.45	0.98-2.15	0.062	1.29	0.72-2.33	0.395
**Model 5**						
urinary sodium (g/d)	1.10	0.95-1.27	0.208	1.17	0.97-1.42	0.111
urinary sodium (g/d)						
(Quartile 2 *vs*. Quartile 1)	0.80	0.53-1.22	0.309	0.96	0.52-1.78	0.900
(Quartile 3 *vs*. Quartile 1)	0.93	0.61-1.39	0.710	0.97	0.52-1.80	0.922
(Quartile 4 *vs*. Quartile 1)	1.31	0.87-1.95	0.194	1.51	0.63-2.11	0.649

DKD, diabetic kidney disease; BMI, body mass index; DBP, diastolic blood pressure; RAS, renin-angiotensin system; HbA1c, glycated hemoglobin; HOMA-IR, homeostasis model assessment of insulin resistance; PWV, pulse wave velocity.

Crude model 1: adjusted for HOMA-IR.

Crude model 2: adjusted for PWV.

Model 1: adjusted for age, gender, BMI, smoking and alcohol consumption.

Model 2: adjusted for model 1+ DBP, RAS blocking agents, diuretics, hyperlipidemia and statin.

Model 3: adjusted for model 2+ HbA1c and antidiabetic medication.

Model 4: adjusted for model 3+ PWV.

Model 5: adjusted for model 4+ HOMA-IR.

## Discussion

In the present study, we provided the evidence regarding the relationship between sodium intake and the presence of DKD in patients with T2DM. Our data showed that high urinary sodium excretion level was significantly associated with UACR level and increased risk of DKD, and exhibited a J-shaped relationship with UACR level. Of note, the relationship between urinary sodium excretion and risks of DKD became insignificant after further adjustment for HOMA-IR and PWV. These findings indicated that dietary sodium was associated with high risks of DKD among patients with T2DM, dependent of vascular sclerosis and insulin resistance.

Our data showed that patients with DKD had higher levels of urinary sodium excretion level than those without DKD. Prior studies showed that a survey in Japanese illustrated a reverse J-shaped relationship between daily salt intake and albuminuria in patients with T2DM (11). In contrast, Horikawa et al. reported no significant difference between sodium intake and the risk of albuminuria in patients with T2DM (12). A small cross-sectional study reported that a high-sodium diet is an independent influencing factor of microalbuminuria and renal dysfunction in 71 patients with T2DM (21). Of note, those studies used dietary recall to calculate sodium intake or estimated from a spot urine sample, which may cause a recall bias, and did not provide conclusive evidence regarding the relationship between sodium intake and the presence of DKD among the patients with T2DM. On the basis of a relative larger sample size, our study estimated sodium intake by urinary sodium excretion, and indicated that high urinary sodium was significantly associated with the risks of UACR level as well as the presence of DKD in patients with T2DM independent of several traditional risk factors. It has been proposed that high sodium intake can increase concentrations of extracellular sodium, and induce myocardial and renal fibrosis (22). Our findings suggest that monitoring sodium intake might be useful for prevention and treatment of DKD in patients with T2DM.

Our data indicated that urinary sodium level was independently correlated with UACR level as well as metabolic risk factors, such as BMI, DBP, SBP, HOMA-IR, and PWV. It has been well documented that several metabolic risk factors, including BMI and blood pressure, play a role in the development of DKD (23–25). Epidemiological study indicated that urinary sodium is linked with incidence of DKD through BMI and blood pressure (26, 27). Vedovato et al. reported that a positive correlation between higher sodium intake and albuminuria in obese adults (28). Overweight and obesity were associated with salt sensitivity and even increase glomerular filtration rate (29). Furthermore, Cardoso and colleague reported that elevated blood pressure was the main predictor of development or progression of DKD in patients with T2DM (30). Our study showed that high urinary sodium excretion was associated with the risk of DKD in patients, even adjusting for BP and BMI.

Interestingly, the relationships between urinary sodium excretion and the risks of DKD and albuminuria were no significant after further adjusting for HOMA-IR and PWV in the present study. These findings indicated that the impact of urinary sodium excretion on DKD may be mediated by other mechanisms, such as vascular sclerosis or insulin resistance. Observational study in Japanese found that individuals with increased PWV was associated with an increased incidence of albuminuria and reduced renal function (31). The proposed mechanisms between arterial stiffness and albuminuria may be involved that increased pulsatile stress from the stiffening of large arteries elevated intrarenal pulse pressure, and lead to microvascular damage and renal insufficiency (32). In addition, insulin resistance is closely correlated with endothelial dysfunction, mild inflammation and oxidative stress, which is involved in the development of arteriosclerosis and DKD (33). Thus, our finding suggests that high urinary sodium excretion is linked with the risks of DKD through increased arteriosclerosis and insulin resistance.

Additionally, our data indicated that 2 g/d of sodium excretion were associated with lower UACR levels, which is consistent with the recommendation by WHO (20). Recently, ADA also recommended a reduction to <2 g/d sodium (5 g/d salt) in patients with diabetes (34). However, guidelines from UK and USA recommended sodium intake reduction that is based on the relationship between high sodium intake and the risks of hypertension and CVD (35, 36). Previous study reported that lower 24-h urinary sodium excretion (<150 mmol Na/day) was inversely associated with increased all-cause mortality (37), which may be higher than the recommended intake (5 g/d salt). Thomas et al. showed that the lowest sodium excretion was associated with the highest cumulative incidence of ESRD in the subgroup of 424 patients with macroalbuminuria (38). Additionally, this study also showed that both high and low sodium intake were associated with adverse mortality outcomes. Our study illustrated that there was a J-shaped relationship between sodium intake and UACR level in patients with T2DM rather than monotonic linear relations. This result also suggested that both higher and lower sodium intake were associated with increased urinary albumin excretion and might cause damage to renal function, underscoring the importance of moderate restriction of sodium intake for reducing the risk of DKD.

This study has the following limitations. First, the study was a cross-sectional design. Causality between urinary sodium excretion and the presence of DKD cannot be determined. It is necessary to determine the association of sodium intake and renal outcomes with long term follow-up period in the prospective cohort study. Second, we used morning fasting urine samples instead of 24 h urine samples for estimating sodium excretion in outpatients.

## Conclusion

In conclusion, our data demonstrated that high urinary sodium excretion was associated with high risk of DKD among patients with T2DM, dependent of vascular sclerosis and insulin resistance. Our findings suggest that moderate restriction of sodium intake might be benefit for reducing the risk of DKD. Further study needs to determine the association of sodium intake and the presence of DKD in the prospective studies.

## Data Availability Statement

The raw data supporting the conclusions of this article will be made available by the authors, without undue reservation.

## Ethics Statement

The study protocol was approved by the Institutional Review Board of Nanfang Hospital of Southern Medical University. The patients/participants provided their written informed consent to participate in this study.

## Author Contributions

YH, HZ, and YX contributed to conception and design of the study. HZ and YX supervised the study. DG, PZ, DL and LY organized the database. YH, JL and Jy L performed the statistical analysis. YH wrote the first draft of the manuscript. YH, WL and JL wrote sections of the manuscript. All authors contributed to the article and approved the submitted version.

## Funding

The National Key Research and Development Project (No.2018YFA0800404); Natural Science Foundation and Key-Area Research and Development Program of Guangdong Province (No.2018B030311031 and 2019B020227004); Clinical Research Startup Program of Southern Medical University by High-level University Construction Funding of Guangdong Provincial Department of Education (No.LC2019ZD010 and 2019CR022); the Medical Scientific Research Foundation of Guangdong Province (No. A2019504); President Foundation of Nanfang Hospital, Southern Medical University (No. 2018C028).

## Conflict of Interest

The authors declare that the research was conducted in the absence of any commercial or financial relationships that could be construed as a potential conflict of interest.

## Publisher’s Note

All claims expressed in this article are solely those of the authors and do not necessarily represent those of their affiliated organizations, or those of the publisher, the editors and the reviewers. Any product that may be evaluated in this article, or claim that may be made by its manufacturer, is not guaranteed or endorsed by the publisher.

## Supplementary Material

Supplementary Figure 1 Odds ratios of DKD in different subgroups. Forest plot of odds ratios of DKD according to urinary sodium excretion levels in different subgroups. Patients were categorized according to the following variables: age (<60 years/≥60 years), gender (male/female), hypertension (yes/no), BMI (< 24 kg/m^2^/≥ 24 kg/m^2^), duration of diabetes (≤ 5 years/> 5 years), HbA1c (< 9%/≥ 9%), SGLT_2i(yes/no), Diuretics(yes/no).
