# Supplementary material for: Association of Urinary Sodium Excretion and Diabetic Kidney Disease in Patients With Type 2 Diabetes Mellitus: A Cross-Sectional Study
Source: Front Endocrinol (Lausanne). 2021 Oct 28;12:772073. doi: 10.3389/fendo.2021.772073 (PMC8582599; doi:10.3389/fendo.2021.772073)
Supplement: Supplementary Figure 1 — Odds ratios of DKD in different subgroups. Forest plot of odds ratios of DKD according to urinary sodium excretion levels in different subgroups. Patients were categorized according to the following variables: age (<60 years/≥60 years), gender (male/female), hypertension (yes/no), BMI (< 24 kg/m2/≥ 24 kg/m2), duration of diabetes (≤ 5 years/> 5 years), HbA1c (< 9%/≥ 9%), SGLT_2i(yes/no), Diuretics(yes/no). [file DataSheet_1.doc]

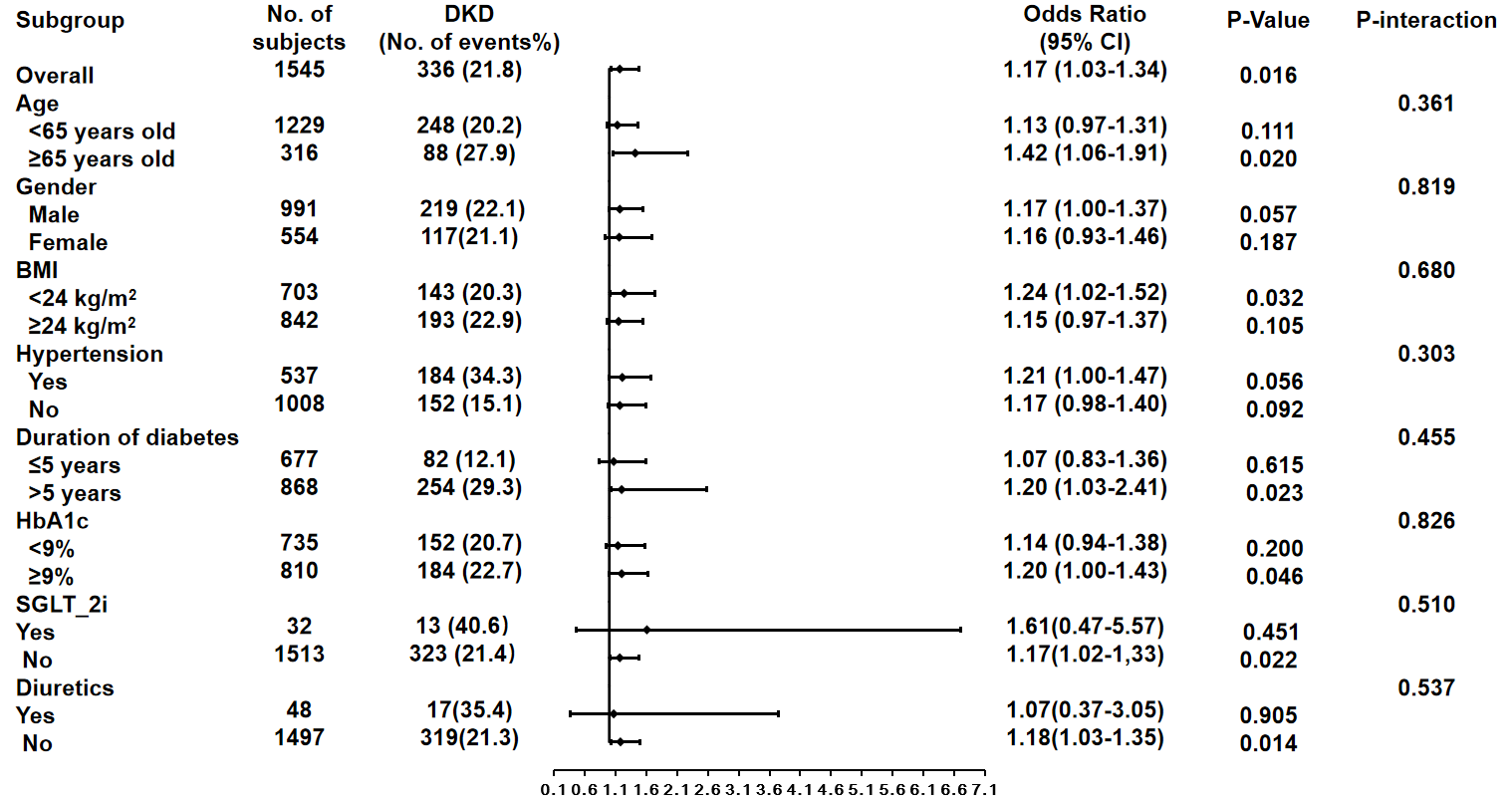


[**SUPPLEMANTARY**](javascript:;) **FIGURE 1**: Forest plot of odds ratios of DKD according to urinary sodium excretion levels in different subgroups. Patients were categorized according to the following variables: age (<60 years/≥60 years), gender (male/female), hypertension (yes/no), BMI (< 24 kg/m2/ ≥ 24 kg/m2), duration of diabetes (≤ 5 years / > 5 years), HbA1c (< 9% / ≥ 9%), SGLT_2i(yes/no), Diuretics(yes/no).
